# Are There Sex Differences in Lower-Limb Biomechanics and Muscle Activation During Rope Jumping in Muay Thai Athletes?

**DOI:** 10.3390/sports13110410

**Published:** 2025-11-13

**Authors:** Torsak Kaewjaratwilai, Niromlee Makaje, Monchai Chottidao

**Affiliations:** 1 Faculty of Education and Development Sciences, Kasetsart University, Kamphaeng Saen Campus, Nakhon Pathom 73140, Thailand; fedutsk@ku.ac.th; 2 Department of Sport Science, Faculty of Sports and Health Science, Kasetsart University, Kamphaeng Saen Campus, Nakhon Pathom 73140, Thailand; niromlee.m@ku.th; 3 College of Sports Science and Technology, Mahidol University, Nakhon Pathom 73170, Thailand

**Keywords:** Muay Thai, muscle activation, vertical leg stiffness, rope jumping

## Abstract

Sex-related differences in lower-limb biomechanics and neuromuscular strategies during rope jumping remain underexplored, particularly in combat-sport athletes. This study investigated leg stiffness and muscle activation in ten female (22.8 ± 0.8 years) and ten male (22.9 ± 1.4 years) Muay Thai athletes. Participants performed rope skipping under three conditions: dominant leg, non-dominant leg, and double leg at 2.2 Hz. Ground reaction forces were recorded at 1000 Hz, center of mass displacement at 200 Hz, and electromyographic activity of the vastus lateralis, biceps femoris, tibialis anterior, and medial gastrocnemius at 3000 Hz. Vertical stiffness (K_vert_) was calculated as the ratio of peak vertical force to displacement. Results showed no significant sex differences in peak ground reaction force (e.g., dominant leg: females 2.83 ± 0.42 vs. males 3.22 ± 0.57 kN; double leg: females 4.04 ± 0.83 vs. males 4.35 ± 0.73 kN; *p* > 0.05), vertical stiffness (females 17.02 ± 3.66 vs. males 16.21 ± 4.09 kN/m; *p* > 0.05), contact time (females 0.280 ± 0.03 vs. males 0.275 ± 0.05 s; *p* > 0.05), or flight time (females 0.205 ± 0.03 vs. males 0.245 ± 0.05 s; *p* > 0.05). In contrast, females exhibited significantly higher co-activation ratios during unilateral skipping, including BF/VL (0.76 ± 0.18 vs. 0.63 ± 0.10; *p* < 0.05) and TA/MG (0.38 ± 0.11 vs. 0.29 ± 0.07; *p* < 0.05), suggesting a neuromuscular strategy to enhance joint stability. These findings highlight rope jumping as a practical drill that can promote neuromuscular control and stability in Muay Thai training.

## 1. Introduction

Leg stiffness (K_leg_) is an important factor in human movement research because it relates to both performance and injury risk [1,2]. Extremely low stiffness increases the chance of muscle damage, while excessively high stiffness raises the risk of bone injury [2,3]. K_leg_ is defined as the ratio of maximal ground reaction force (Max_GRF_) to the vertical displacement of the center of mass during stance. In simple terms, stiffness reflects the relationship between the force applied and the body’s deformation. During running, hopping, and jumping, the lower limbs act like a spring system, where body mass is supported by leg stiffness [4]. Athletes with higher K_leg_ can generally run faster and jump higher because they transfer energy more efficiently.

Previous studies have examined gender differences in stiffness and muscle activation. For example, Padua et al. (2005) reported higher quadriceps-to-hamstring co-activation ratios in females but no significant gender differences in stiffness at 3.0 Hz hopping [5]. Similarly, Hobara et al. (2012) found a positive relationship between passive ankle stiffness and leg stiffness in females, though not significantly different between sexes [6]. Brauner (2014) observed no difference in stiffness between dominant and non-dominant legs in unilateral hopping [7]. Furthermore, leg stiffness has been positively correlated with sprint velocity [6,8,9]. While these findings provide important insights from general athletic populations, they do not capture the sport-specific neuromuscular demands of Muay Thai. Rope jumping in Muay Thai athletes involves both repetitive unilateral kicking and bilateral footwork, creating a unique context to investigate whether established gender-related activation patterns extend to combat sports. This study therefore addresses a gap by examining rope skipping in a combat-sport population, providing new perspectives on neuromuscular control beyond what has been reported in general athletes.

Ground contact time is another important factor: higher hopping frequency typically increases stiffness while reducing contact time [10,11]. Studies of drop jumps at different heights showed shorter contact times and higher stiffness with increasing jump height [12]. Hobara et al. (2015) compared younger and older participants and found similar spring-mass behavior across hopping frequencies, though fatigue protocols decreased stiffness due to reduced joint moment and muscle activation [13]. Other research has shown no clear effect of leg dominance, running speed, or sidedness on stiffness [14,15]. In particular, hopping frequencies around 2.2 Hz have been frequently adopted in previous studies to provide a stable and representative condition for assessing leg stiffness, as shown by Hobara et al. (2013), who examined stiffness at 1.5, 2.2, and 3.0 Hz and identified 2.2 Hz as a suitable reference point for evaluating bilateral differences [16].

In addition, muscle activation patterns differ with contact phases. Short contact time phases are associated with higher stiffness and greater activation of the gastrocnemius and soleus [17]. Training studies show that endurance runners typically present lower stiffness than power-trained athletes, reflecting adaptations in neuromuscular control [18]. Jumping rope, or skipping, is a simple yet effective training method to improve fitness, coordination, balance, and cardiovascular endurance. It is widely used in sports such as track and field, boxing, basketball, and martial arts [19]. Rope jumping involves repetitive stretch-shortening cycle (SSC) actions of the lower limbs, similar to plyometric training [20]. Studies have compared energy expenditure, lactate responses, and cardiovascular adaptations, showing rope skipping to be efficient and beneficial [21,22,23]. Variations such as double-unders further enhance SSC demands and sprint performance [20]. Moreover, rope skipping improves neuromuscular coordination, with trained individuals displaying shorter EMG activation times compared to untrained groups [14]. Despite these findings, research on leg stiffness during rope jumping is still limited, especially regarding gender differences. Most evidence comes from running, hopping, and drop jumps, leaving rope skipping underexplored.

Therefore, the purpose of this study is to examine sex differences in leg stiffness and muscle activation during unilateral and bilateral rope jumping among Muay Thai athletes. This rationale is based on evidence that muscle mass and force production capacity may partly explain sex-related differences in stiffness and neuromuscular strategies, yet prior studies have not examined these mechanisms in the context of rope skipping. We hypothesize that male athletes will demonstrate greater leg stiffness than female athletes, reflecting differences in muscle mass and force production. Furthermore, we expect dominant legs to show higher stiffness than non-dominant legs due to repeated kicking and loading patterns in training and competition. In terms of muscle activation, female athletes are anticipated to present higher quadriceps-to-hamstring co-activation ratios, whereas male athletes will exhibit greater gastrocnemius and soleus activation. The objective is therefore stated as a single, clearly defined aim to address this gap, avoiding redundancy with other sections.

## 2. Materials and Methods

### 2.1. Participants

A total of twenty Muay Thai athletes were recruited using purposeful sampling from the National Sports University (females, *n* = 10; age 22.80 ± 0.78 yr, body weight 60.70 ± 7.49 kg, height 161.60 ± 7.93 cm; males, *n* = 10; age 22.90 ± 1.37 yr, body weight 63.90 ± 9.98 kg, height 172.30 ± 3.46 cm). All participants had at least three years of Muay Thai training experience and practiced a minimum of four days per week.

Inclusion criteria were: (1) a minimum of three years of Muay Thai training; (2) training at least four days per week; (3) no musculoskeletal injuries within the past three months; and (4) no history of serious lower-limb injuries or surgeries (e.g., ACL injury, fracture, patellar dislocation). Exclusion criteria were: (1) current injury or pain affecting performance; (2) history of neurological disorders; and (3) use of ergogenic aids or dietary supplements that could influence rope skipping performance. Participants confirmed that they did not use any ergogenic aids or supplements during the study.

Participant eligibility was confirmed using a purpose-designed questionnaire assessing training background and physical activity level. The study protocol was approved by the Human Ethics Committee, and written informed consent was obtained from all participants before the experiment.

An a priori power analysis was conducted in G*Power 3.1 [24] for a mixed design with one between-subjects factor (sex: male vs. female) and one within-subjects factor (task: unilateral vs. bilateral rope jumping; 2 levels). The analysis assumed a medium effect size for the sex × task interaction (f = 0.25), with α = 0.05, power (1−β) = 0.80, correlation among repeated measures r = 0.50, and nonsphericity correction ε = 1.0. Based on these parameters, the required total sample size was N = 18. Allowing for ~10% attrition, we targeted N = 20 (10 per sex), which was achieved. In the subsequent statistical analysis, both main effects and the sex × task interaction were tested and reported accordingly.

### 2.2. Procedure

Participants performed barefoot rope jumping on a force platform under three conditions: non-dominant leg (NDL), dominant leg (DL), and double-leg (DOL). The dominant leg was defined as the preferred leg used to kick a ball, whereas the non-dominant leg was identified as the supporting leg. Each condition was performed at a controlled frequency of 2.2 Hz (132 jumps per minute), guided by a digital metronome to ensure consistent cadence. Each trial lasted 30 s, and the order of conditions was randomized across participants. A standardized 2 min passive rest interval was provided between conditions to minimize fatigue. This rest duration was adopted based on previous plyometric and jump performance studies, which demonstrated that a 2 min recovery period is sufficient to restore performance capacity and minimize carry-over fatigue between trials [25,26,27].

The frequency of 2.2 Hz was selected because previous studies have demonstrated that this cadence elicits stable spring–mass behavior and is widely used to evaluate leg stiffness during hopping tasks [13,16]. This frequency allows for reliable comparisons across conditions while minimizing variability in ground contact time and flight time.

### 2.3. Kinetic Data Collection and Analysis

Kinetic data during rope jumping were collected using a force platform (AMTI, Inc., Newton, MA, USA) at a sampling frequency of 1000 Hz. Each trial lasted 30 s, and five consecutive jumps (6th to 10th) were selected for analysis. The first 1–4 s of each trial were excluded to avoid variability during the acceleration phase, and jumps after the 10th cycle were not analyzed to minimize potential fatigue effects. Therefore, the 6th–10th cycles were chosen to represent steady-state performance while controlling for both initial adaptation and later fatigue. Ground reaction force (GRF) recordings were used to determine ground contact time (tc), flight time (tf), and to compute leg stiffness (K_leg_) based on the spring–mass model.

Leg stiffness was calculated using the spring–mass model [4]. Stiffness was defined as the ratio of peak vertical ground reaction force to the vertical displacement of the center of mass (COM) between its lowest position at mid-stance and its highest position during flight [28]. Leg stiffness (K_leg_) was defined in this study as the ratio of peak vertical ground reaction force to the vertical displacement of the center of mass (COM). For clarity, the term vertical stiffness (Kvert) is sometimes used in the literature with a similar definition; however, in the present study we consistently use Kleg to denote leg stiffness derived from the spring–mass model.

K_leg_ = F_max_/Δy

where F_max_ is the peak vertical ground reaction force, and Δy is the vertical displacement of the center of mass.

COM displacement was obtained from body markers recorded with ten high-speed cameras (200 Hz; Motion Analysis System, Santa Rosa, CA, USA). A total of 29 reflective markers were placed according to the Helen Hayes marker set to define the segmental model. Markers were attached to anatomical landmarks including the head (top, front, rear), bilateral shoulders and offset, elbows, wrists, anterior superior iliac spines (ASIS), sacrum, thighs, medial and lateral knees, shanks, medial and lateral ankles, heels, and toes. Marker trajectories were processed using Orthotrak software (version 6.2.4; Single Trial Processing Module (version 3.2); Motion Analysis System, Vicon Nexus (version 2.14); Clinical Gait Analysis Software (version 4.0)) to compute COM displacement. Raw marker data were low-pass filtered at 6 Hz using a fourth-order Butterworth filter, which is commonly applied in gait and jump analyses to reduce high-frequency noise while preserving movement signals [29,30]. The global coordinate system was defined with the x-axis oriented anterior–posterior, y-axis medial–lateral, and z-axis vertical, ensuring reproducibility of COM calculations across participants.

### 2.4. EMG Collection and Analysis

Maximal voluntary contraction (MVC) testing was performed using a Biodex System 4 Pro Dynamometer (Biodex Medical Systems, Shirley, NY, USA) to normalize EMG signals. Participants performed maximal isometric flexion and extension of the knee joint and dorsiflexion and plantarflexion of the ankle joint for 5 s each, with both joints positioned at 90°.

Electromyographic activity (EMG) was recorded using a wireless telemetry system (TeleMyo 2400T, Noraxon, Scottsdale, AZ, USA) from the vastus lateralis (VL), biceps femoris (BF), tibialis anterior (TA), and medial gastrocnemius (MG) of both legs. Prior to electrode placement, the skin was shaved, abraded, and cleaned with alcohol wipes to reduce impedance. Disposable surface electrodes (Noraxon USA Inc., Scottsdale, AZ, USA) were positioned over the muscle belly according to the SENIAM guidelines [31]. EMG signals were pre-amplified, band-pass filtered at 15–1000 Hz, and sampled at 3000 Hz following established recommendations for surface EMG acquisition [32,33]. Normalized EMG activity (%MVC) was calculated as the mean and standard deviation across conditions and rope-jumping phases.

Muscle activity was analyzed in different temporal phases relative to ground contact: (1) pre-activation (PRE), defined as the mean EMG within 100 ms before landing; (2) background activity (BGA), defined as the first 30 ms after foot contact; (3) short-latency reflex (M1), defined as EMG activity 30–60 ms post-contact; and (4) long-latency reflex (M2), defined as EMG activity 60–90 ms post-contact [17,18]. M1 and M2 windows were specifically selected because they correspond to well-established neuromuscular reflex responses following stretch-shortening cycle actions, allowing for standardized comparison across studies, whereas task-specific phases such as stance or flight may vary depending on cadence and individual technique.

Co-activation ratios were also calculated to assess joint stability. At the knee, the ratio of BF to VL represented hamstring–quadriceps co-activation, while at the ankle, the ratio of TA to MG represented dorsiflexor–plantarflexor co-activation.

### 2.5. Statistical Analysis

All statistical analyses were performed using SPSS software (version 20.0; IBM Corp., Armonk, NY, USA). Data normality was verified using the Kolmogorov–Smirnov test. Independent t-tests were applied to compare kinetic variables between male and female Muay Thai athletes. Paired t-tests were conducted to examine differences between measurement methods for vertical leg stiffness and center of mass displacement. Differences across jumping conditions (NDL, DL, and DOL) were analyzed using one-way repeated-measures ANOVA. In addition, a two-way mixed-design ANOVA was employed with sex (male vs. female) as the between-subjects factor and condition (NDL, DL, DOL) as the within-subjects factor, followed by Bonferroni-adjusted pairwise comparisons to control for multiple testing and reduce the risk of Type I error. Mauchly’s test of sphericity was applied to assess the assumption of sphericity, and when violated, Greenhouse–Geisser corrections were used. Effect sizes were also calculated, with partial eta squared (η^2^) reported for ANOVA results and Cohen’s d for pairwise comparisons, to enhance interpretation given the small sample size. Statistical significance was set at *p* < 0.05.

## 3. Results

### 3.1. Peak Ground Reaction Force, Contact Time, and Flight Time

Peak ground reaction force (GRF_Peak_) values were lower in Muay Thai females compared with males across all jumping conditions, although these differences were not statistically significant (*p* > 0.05). Within each sex, GRF_Peak_ was significantly lower during unilateral (dominant and non-dominant leg) rope jumping than during bilateral rope jumping (*p* < 0.05). The corresponding effect sizes indicated large magnitudes of difference between jumping modes (females: η^2^ = 0.46, d = 1.84; males: η^2^ = 0.43, d = 1.73), reflecting substantial increases in ground reaction forces under double-leg conditions.

No significant main effect of sex was observed for contact time (*p* > 0.05). However, contact time during double-leg rope jumping was significantly shorter than during unilateral rope jumping in both sexes (*p* < 0.05). The observed effect sizes demonstrated large effects (females: η^2^ = 0.50, d = 2.00; males: η^2^ = 0.29, d = 1.26), confirming meaningful reductions in ground contact duration during the double-leg condition.

Flight time also showed no significant differences between sexes (*p* > 0.05). Nevertheless, flight time was significantly longer during double-leg compared with unilateral rope jumping within both groups (*p* < 0.05). The corresponding effect sizes were large (females: η^2^ = 0.27, d = 1.21; males: η^2^ = 0.19, d = 0.97), indicating that the bilateral condition induced greater airtime and rebound performance (Table 1).

### 3.2. Leg Stiffness (K_leg_) and Center of Mass Displacement (COM)

Leg stiffness (K_leg_) defined as the ratio of peak ground reaction force to the vertical displacement of the center of mass, did not differ significantly between Muay Thai males and females across all jumping conditions (*p* > 0.05). However, within both sex groups, K_leg_ values were significantly higher during double-leg rope jumping compared with unilateral conditions (*p* < 0.05). The corresponding effect sizes indicated small-to-moderate magnitudes (females: η^2^ = 0.09, d = 0.62; males: η^2^ = 0.13, d = 0.76), suggesting that although statistically significant, the practical differences in stiffness were modest.

Center of mass displacement (COM) followed a similar pattern, showing greater vertical motion during the double-leg condition compared with unilateral jumping in both sexes (*p* < 0.05). These findings collectively indicate that bilateral rope jumping requires greater vertical impulse and elastic energy utilization, while unilateral conditions rely more on rapid stiffness and shorter contact control (Table 1).

### 3.3. Electromyographic Activity

Electromyographic (EMG) responses revealed distinct activation patterns across the four analyzed lower-limb muscles. The medial gastrocnemius (MG) and vastus lateralis (VL) exhibited the highest overall activity, whereas the tibialis anterior (TA) demonstrated the lowest amplitudes across all contraction phases—from pre-activation (PRE) to the long-latency stretch reflex (M2). Peak activation occurred during the short-latency reflex phase (M1, 30–60 ms post-landing), reflecting rapid muscle response within the stretch–shortening cycle.

Overall, Muay Thai females displayed slightly greater EMG amplitudes than males in all examined muscles (VL, BF, TA, MG), with some reaching statistical significance (*p* < 0.05) (Table 2). For example, VL activation during M1 was significantly higher in females (η^2^ = 0.31, d = 0.86), and MG activation during BGA and M1 phases also showed large effects (η^2^ = 0.38–0.40, d ≈ 1.0), indicating stronger reflexive activation and neuromuscular responsiveness in female athletes.

No significant differences were found between dominant and non-dominant legs for any muscle across phases in either sex (*p* > 0.05), with small-to-moderate effect sizes (η^2^ < 0.25, d < 0.75), suggesting symmetrical neuromuscular control. However, within females, double-leg rope jumping produced slightly higher activation in the PRE and M2 phases (η^2^ ≈ 0.33, d ≈ 0.9) compared with unilateral conditions (*p* < 0.05), indicating enhanced pre-tension and post-impact stabilization when both limbs were involved.

Regarding muscle co-activation, the BF/VL and TA/MG ratios were significantly higher in Muay Thai females than in males during both PRE and M2 phases (*p* < 0.05, d = 0.48–0.63) (Table 3). These moderate-to-large effect sizes reflect greater agonist–antagonist coordination in females, potentially contributing to enhanced joint stability and controlled landing mechanics during rope jumping.

## 4. Discussion

The objective of this study was to examine leg stiffness and muscle activation during unilateral and bilateral rope jumping in Muay Thai athletes. EMG amplitudes in VL, BF, TA, and MG were generally higher in females than in males, with some differences reaching statistical significance. This suggests that female athletes performed rope jumping with greater muscle activation. Consistent with previous findings, females have been reported to recruit quadriceps activation up to twice as much as males during double-leg hopping, partly due to lower body mass and the need to adjust vertical leg stiffness [5]. However, higher activation may not necessarily indicate greater effort; it could also reflect compensatory or less efficient neuromuscular strategies, which should be considered when interpreting these findings.

No significant differences were found between dominant and non-dominant legs in muscle activation or leg stiffness. This suggests that both legs contribute similarly to stability and load absorption during rope jumping, likely due to balanced neuromuscular adaptations developed through Muay Thai training. Such adaptations may enable athletes to use both the kicking and supporting legs interchangeably to maintain joint stability and absorb impact forces. This observation is consistent with a previous study on basketball landing tasks, which reported comparable muscle co-activation patterns across legs [34]. Similar findings have also been reported in running [35] and Taekwondo athletes [36], where no significant asymmetry between dominant and non-dominant legs was observed.

Importantly, the co-activation ratios (BF/VL, TA/MG) during unilateral rope jumping were significantly higher in female Muay Thai athletes compared to their male counterparts, both before and after landing. This finding indicates that female athletes may adopt a neuromuscular strategy to enhance joint stability during both preparatory and impact phases of take-off and landing. Such an adaptation is consistent with previous reports showing that females rely more on quadriceps and gastrocnemius activation while exhibiting reduced hamstring co-contraction [5,37,38]. Within the context of Muay Thai, this heightened co-activation may function as a protective mechanism against lower-limb injuries during high-frequency rope skipping and plyometric training, while also contributing to balance and defensive stability during combat situations.

Regarding leg stiffness (K_leg_), no significant differences were detected between female and male Muay Thai athletes across all rope jumping conditions. This finding aligns with prior studies reporting no sex-related differences in stiffness during hopping at controlled frequencies [6]. Although some earlier research suggested that women may demonstrate lower stiffness compared with men [3], body mass and anthropometric characteristics are likely to be influential factors. In the present study, a statistical comparison of body composition between sexes was not conducted; therefore, the findings should be interpreted with caution and acknowledged as a limitation. Furthermore, we did not perform a correlation analysis between body mass and K_leg_, nor did we normalize stiffness values to body mass. These approaches may provide additional insight and should be considered in future studies.

Similarly, no differences in K_leg_ were found between dominant and non-dominant legs. This agrees with previous reports of equivalent stiffness between legs during unilateral hopping tasks at multiple frequencies [7,16]. One explanation may be that the moderate intensity of 2.2 Hz used in this study was not sufficient to induce asymmetries. For Muay Thai athletes, this result suggests that rope jumping at a standardized frequency engages both legs symmetrically, regardless of kicking dominance.

In summary, this study showed that Muay Thai females demonstrated greater muscle activation and higher co-activation ratios than males during rope skipping, reflecting sex-specific neuromuscular strategies. In contrast, no sex or limb differences were observed in leg stiffness, suggesting symmetrical contributions of both legs at the tested cadence. Practically, rope jumping at 2.2 Hz (≈130 jumps/min) can be prescribed at moderate intensity for 3–5 sets of 30–60 s with 1–2 min rest to improve neuromuscular control, enhance lower-limb conditioning, and support joint stability. These findings provide coaches with evidence-based guidance for applying rope skipping in training programs aimed at injury prevention and performance optimization, particularly for female athletes.

## 5. Conclusions

Muay Thai females showed greater overall muscle activation and higher co-activation ratios than males during rope skipping, indicating a distinct neuromuscular strategy that may contribute to joint stability. In contrast, no significant sex or limb differences were found in leg stiffness, suggesting that both legs contributed symmetrically under the tested condition with limited biomechanical variables. The novelty of this study lies in demonstrating that sex differences emerge at the neuromuscular level rather than in external biomechanics. These findings highlight rope skipping not only as a fundamental conditioning exercise but also as a valuable tool for improving rhythm, footwork, and neuromuscular control, while potentially serving as a strategy for injury prevention and performance optimization in both male and female Muay Thai athletes.

### Limitations and Future Directions

This study has several limitations that should be acknowledged when interpreting the findings. First, the relatively small sample size (n = 20) limits the statistical power and generalizability of the results to broader populations of Muay Thai athletes or other combat sports. Second, although EMG recordings provide insights into neuromuscular strategies, surface EMG is inherently subject to variability due to electrode placement, cross-talk, and skin impedance. Third, rope skipping was examined at a single cadence of 2.2 Hz, which, while commonly adopted in prior literature, does not represent the range of frequencies encountered in training and competition. Minor discrepancies between the prescribed cadence and calculated contact–flight cycle durations further highlight the need for caution in cycle-based interpretations. Fourth, body composition and anthropometric variables were not statistically compared between sexes, which may partly explain stiffness differences and should be addressed in future work.

Future studies should therefore include larger and more diverse cohorts, incorporate multiple rope skipping frequencies and task conditions, and employ longitudinal or interventional designs to evaluate causal relationships with performance and injury risk. Integrating more comprehensive biomechanical analyses (e.g., joint moments, kinematics) together with normalized stiffness measures may further clarify sex-specific adaptations. Such approaches will enhance the translational value of rope skipping research for training design, athlete monitoring, and injury-prevention strategies in combat sports.

## 6. Practical Implications

### 6.1. Rope Skipping as Joint Stability Training

The higher co-activation ratios observed in Muay Thai females suggest that rope jumping may provide a useful approach to practice neuromuscular control and joint stability, particularly at the knee and ankle, within the limits of a cross-sectional design. Rather than establishing direct injury-prevention effects, these findings could inform future longitudinal or interventional studies aimed at evaluating rope skipping as part of athlete development programs. Coaches may consider incorporating rope skipping drills as a complementary exercise, while acknowledging that evidence for direct injury-prevention benefits remains limited and requires further investigation.

### 6.2. Frequency and Intensity Monitoring

Although cadence was controlled using a digital metronome set at 2.2 Hz, slight discrepancies were observed between the prescribed frequency and the average contact + flight times. These differences likely reflect natural variability in execution and averaging procedures rather than true deviations from the target cadence. Nevertheless, the possibility of minor inconsistencies in actual jumping frequency should be acknowledged as a limitation, as it may have influenced cycle-based interpretations.

### 6.3. Skill Transfer to Muay Thai Performance

Enhanced joint stability and neuromuscular coordination developed through rope jumping may translate to improved footwork, balance, and defensive control in Muay Thai. This is especially relevant during rapid transitions between offensive and defensive movements in the ring.

### 6.4. Gender-Specific Training Considerations

Given the tendency of Muay Thai females to exhibit greater muscle co-activation, coaches may tailor training to build efficiency and reduce unnecessary muscular effort, while for Muay Thai males, emphasis may be placed on improving co-activation for added stability.

### 6.5. Integration with Periodization

Rope jumping can be strategically incorporated into periodized training programs not only as a warm-up or conditioning drill but also as a targeted plyometric exercise to reinforce stretch–shortening cycle adaptations and sport-specific endurance.

## Figures and Tables

**Table 1:** Kinetic characteristics of rope jumping, including peak ground reaction force (GRFpeak), leg stiffness (Kleg), contact time (tc), and flight time (tf), across dominant-leg, non-dominant-leg, and double-leg jumping conditions. Effect sizes (η^2^ and Cohen’s d) are presented to indicate the magnitude of differences between conditions.

Parameter	Sex	Dominant Leg	Non-Dominant Leg	Double Leg	Effect Size (η^2^/Cohen’s d)
GRF_Peak_ (kN)	Females	2.83 ± 0.42	2.96 ± 0.60	4.04 ± 0.83 ^a,b^	η^2^ = 0.46 (d = 1.84)
	Males	3.22 ± 0.57	3.33 ± 0.45	4.35 ± 0.73 ^a,b^	η^2^ = 0.43 (d = 1.73)
K_leg_ (kN/m)	Females	17.02 ± 3.66	16.47 ± 3.61	19.88 ± 5.46	η^2^ = 0.09 (d = 0.62)
	Males	16.21 ± 4.09	16.58 ± 3.38	19.08 ± 3.44	η^2^ = 0.13 (d = 0.76)
tc (s)	Females	0.280 ± 0.03	0.257 ± 0.04	0.220 ± 0.03 ^a,b^	η^2^ = 0.50 (d = 2.00)
	Males	0.275 ± 0.05	0.279 ± 0.04	0.223 ± 0.03 ^a,b^	η^2^ = 0.29 (d = 1.26)
tf (s)	Females	0.205 ± 0.03	0.206 ± 0.04	0.255 ± 0.05 ^a,b^	η^2^ = 0.27 (d = 1.21)
	Males	0.245 ± 0.05	0.224 ± 0.03	0.285 ± 0.03 ^a,b^	η^2^ = 0.19 (d = 0.97)

GRF_peak_ = peak vertical ground reaction force; K_leg_ = leg stiffness; tc = contact time; tf = flight time. ^a^ Significant difference between dominant and double-leg conditions (*p* < 0.05). ^b^ Significant difference between non-dominant and double-leg conditions (*p* < 0.05). Effect sizes are reported as partial eta squared (η^2^) for the ANOVA and Cohen’s d for pairwise comparisons.

**Table 2:** Electromyographic activity and effect sizes (η^2^, d) of lower-limb muscles during rope jumping across conditions.

Muscle	Sex	Condition	PRE (µV)	BGA (µV)	M1 (µV)	M2 (µV)	Effect Size (η^2^/d)
Vastus lateralis	Male	Dominant	71.92 ± 7.23	62.03 ± 12.62	66.48 ± 10.13	62.50 ± 5.44	η^2^ = 0.35/d = 0.92
		Non-dominant	71.24 ± 11.08	66.13 ± 5.59	68.25 ± 3.16	68.75 ± 8.73	η^2^ = 0.33/d = 0.90
		Double	52.27 ± 9.12	58.62 ± 10.12	59.56 ± 11.61	56.36 ± 10.39	η^2^ = 0.33/d = 0.90
	Female	Dominant	73.46 ± 9.26	70.29 ± 5.80	70.71 ± 5.79 *	69.62 ± 11.50	η^2^ = 0.31/d = 0.86
		Non-dominant	69.68 ± 11.63	62.92 ± 10.84	67.72 ± 11.67 *	71.80 ± 13.75	η^2^ = 0.33/d = 0.90
		Double	58.96 ± 17.04 ^a^	65.00 ± 21.61 *	67.87 ± 24.79 *	67.82 ± 28.36 *^b^	η^2^ = 0.33/d = 0.90
Biceps femoris	Male	Dominant	44.76 ± 4.45	42.78 ± 7.97	42.00 ± 8.29	42.33 ± 11.23	η^2^ = 0.26/d = 0.74
		Non-dominant	42.86 ± 7.91	48.96 ± 7.02	52.03 ± 6.76	45.16 ± 5.38	η^2^ = 0.25/d = 0.72
		Double	42.13 ± 12.30	40.96 ± 13.43	41.19 ± 14.20	39.37 ± 16.41	η^2^ = 0.25/d = 0.72
	Female	Dominant	55.37 ± 10.57 *	51.51 ± 5.80	54.26 ± 5.94	59.79 ± 12.36	η^2^ = 0.23/d = 0.68
		Non-dominant	50.26 ± 12.20	49.65 ± 9.50	51.85 ± 10.48	55.53 ± 11.64 *	η^2^ = 0.25/d = 0.72
		Double	38.85 ± 9.20	49.86 ± 13.09	62.22 ± 31.34	71.00 ± 43.85	η^2^ = 0.25/d = 0.72
Tibialis anteriors	Male	Dominant	27.27 ± 3.76	27.31 ± 5.47	29.82 ± 4.18	33.91 ± 7.99	η^2^ = 0.19/d = 0.61
		Non-dominant	26.93 ± 5.22	30.08 ± 11.02	29.16 ± 8.12	28.08 ± 6.89	η^2^ = 0.18/d = 0.60
		Double	29.23 ± 6.02	31.06 ± 4.86	33.19 ± 6.72	31.30 ± 8.64	η^2^ = 0.18/d = 0.60
	Female	Dominant	35.31 ± 11.71	32.32 ± 12.65	32.08 ± 12.57 *	32.68 ± 15.74 *	η^2^ = 0.16/d = 0.57
		Non-dominant	30.47 ± 10.54	29.61 ± 7.79	27.93 ± 6.86	28.53 ± 9.39	η^2^ = 0.18/d = 0.60
		Double	26.95 ± 10.26	27.43 ± 9.12	27.99 ± 8.62	26.92 ± 8.19	η^2^ = 0.18/d = 0.60
Medial gastrocnemius	Male	Dominant	93.68 ± 13.12	96.93 ± 3.57	102.14 ± 4.44	78.00 ± 2.77	η^2^ = 0.42/d = 1.05
		Non-dominant	94.06 ± 5.38	95.10 ± 12.03	97.24 ± 14.14	78.82 ± 6.23	η^2^ = 0.40/d = 1.02
		Double	70.09 ± 5.53	80.04 ± 6.76	81.65 ± 7.22	69.43 ± 7.79	η^2^ = 0.40/d = 1.02
	Female	Dominant	91.42 ± 7.47	100.81 ± 11.51 *	108.59 ± 14.48 *	87.82 ± 22.17 *	η^2^ = 0.38/d = 0.98
		Non-dominant	91.25 ± 7.06	98.33 ± 13.04	105.84 ± 14.34	89.63 ± 20.61	η^2^ = 0.40/d = 1.02
		Double	74.96 ± 11.99 *^a,b^	88.01 ± 13.92	100.57 ± 17.97	98.69 ± 23.77 *^a,b^	η^2^ = 0.40/d = 1.02

Values are presented as mean ± SD. PRE = pre-activation; BGA = background activity; M1 = short-latency stretch reflex; M2 = long-latency stretch reflex. Effect sizes are expressed as partial eta squared (η^2^) for ANOVA and Cohen’s d for pairwise comparisons. * Significant difference between males and females within the same condition (*p* < 0.05). ᵃ Significant difference between dominant and double-leg conditions (*p* < 0.05). ᵇ Significant difference between non-dominant and double-leg conditions (*p* < 0.05).

**Table 3:** Co-activation indices (BF/VL and TA/MG) during rope jumping in male and female Muay Thai athletes.

Phase	Sex	Condition	BF/VL (Mean ± SD)	TA/MG (Mean ± SD)	Effect Size (d)
Pre-activity (PRE)	Male	Dominant	0.63 ± 0.10	0.29 ± 0.07	d = 0.45
	Female		0.76 ± 0.18 *	0.38 ± 0.11	d = 0.52
	Male	Non-dominant	0.62 ± 0.22	0.28 ± 0.06	d = 0.38
	Female		0.74 ± 0.22	0.33 ± 0.11	d = 0.48
	Male	Double	0.80 ± 0.16	0.42 ± 0.10	d = 0.55
	Female		0.68 ± 0.15	0.37 ± 0.19	d = 0.49
Background activity (BGA)	Male	Dominant	0.70 ± 0.14	0.28 ± 0.06	d = 0.40
	Female		0.73 ± 0.11	0.31 ± 0.09	d = 0.46
	Male	Non-dominant	0.74 ± 0.12	0.33 ± 0.15	d = 0.42
	Female		0.83 ± 0.33	0.30 ± 0.07	d = 0.47
	Male	Double	0.70 ± 0.21	0.39 ± 0.06	d = 0.52
	Female		0.80 ± 0.17	0.31 ± 0.12	d = 0.55
Short-latency stretch reflex (M1)	Male	Dominant	0.63 ± 0.13	0.29 ± 0.04	d = 0.42
	Female		0.77 ± 0.12	0.29 ± 0.10	d = 0.48
	Male	Non-dominant	0.76 ± 0.10	0.31 ± 0.12	d = 0.44
	Female		0.81 ± 0.34	0.26 ± 0.07	d = 0.46
	Male	Double	0.70 ± 0.27	0.40 ± 0.07	d = 0.56
	Female		0.92 ± 0.29	0.28 ± 0.11	d = 0.60
Long-latency stretch reflex (M2)	Male	Dominant	0.68 ± 0.19	0.43 ± 0.10	d = 0.47
	Female		0.87 ± 0.24 *	0.40 ± 0.22 *	d = 0.55
	Male	Non-dominant	0.66 ± 0.11	0.36 ± 0.10	d = 0.42
	Female		0.81 ± 0.28 *	0.32 ± 0.11	d = 0.51
	Male	Double	0.70 ± 0.30	0.45 ± 0.11	d = 0.58
	Female		1.02 ± 0.38	0.28 ± 0.10	d = 0.63

* Significant difference between males and females within the same condition (*p* < 0.05).

## Data Availability

Data are not publicly available due to privacy and ethical restrictions. The datasets generated and analyzed during the current study are available from the corresponding author on reasonable request.
